# Complement receptor 3 mediates *Aspergillus fumigatus* internalization into alveolar epithelial cells with the increase of intracellular phosphatidic acid by activating FAK

**DOI:** 10.1080/21505594.2021.1958042

**Published:** 2021-08-02

**Authors:** Xuelin Han, Xueting Su, Zhiqian Li, Yanxi Liu, Shuo Wang, Miao Zhu, Changjian Zhang, Fan Yang, Jingya Zhao, Xianping Li, Fangyan Chen, Li Han

**Affiliations:** a Department for Disinfection and Infection Control, Chinese PLA Center for Disease Control and Prevention, Beijing, China; b Department of Laboratory Medicine & Blood Transfusion, the 907th Hospital, Fujian, Nanping, China; c Northwest Institute of Plateau Biology, Chinese Academy of Science, Qinghai, Xining, China; d Central Laboratory of the sixth medical center of PLA general hospital, Beijing, China; e State Key Laboratory for Infectious Disease Prevention and Control, National Institute for Communicable Disease Control and Prevention, Chinese Center for Disease Control and Prevention, Beijing, China

**Keywords:** *Aspergillus fumigatus*, Complement receptor 3, alveolar epithelial cells, opsonization, phosphatidic acid, FAK

## Abstract

Complement receptor 3 (CD11b/CD18) is an important receptor that mediates adhesion, phagocytosis and chemotaxis in various immunocytes. The conidia of the medically-important pathogenic fungus, *Aspergillus fumigatus* can be internalized into alveolar epithelial cells to disseminate its infection in immunocompromised host; however, the role of CR3 in this process is poorly understood. In the present study, we investigated the potential role of CR3 on *A. fumigatus* internalization into type II alveolar epithelial cells and its effect on host intracellular PA content induced by *A. fumigatus*. We found that CR3 is expressed in alveolar epithelial cells and that human serum and bronchoalveolar lavage fluid (BALF) could improve *A. fumigatus* conidial internalization into A549 type II alveolar epithelial cell line and mouse primary alveolar epithelial cells, which were significantly inhibited by the complement C3 quencher and CD11b-blocking antibody. Serum-opsonization of swollen conidia, but not resting conidia led to the increase of cellular phosphatidic acid (PA) in A549 cells during infection. Moreover, both conidial internalization and induced PA production were interfered by CD11b-blocking antibody and dependent on FAK activity, but not Syk in alveolar epithelial cells. Overall, our results revealed that CR3 is a critical modulator of *Aspergillus fumigatus* internalization into alveolar epithelial cells.

## Introduction

*Aspergillus fumigatus* is a ubiquitous opportunistic pathogen with airborne conidia, which is small enough (diameter of 2–3 μm) to be inhaled into human airways and even deeply embedded into the lung alveoli. In the immunocompromised host, inhaled conidia could lead to induce a lethal invasive infection, and in some cases, even death [1]. Previous studies on the interactions of *A. fumigatus* with host innate immune system mostly focused on professional immune cells, such as macrophages, neutrophils and monocytes; however, increasing number of studies indicated that lung epithelial cells, as the initial contact point between the airborne pollutant and the host, act not only as a physical barrier but also a critical player in the host innate immunity against *A. fumigatus* [2].

The conidia of *A. fumigatus* may adhere to and be internalized into lung epithelial cells, thus inducing the release of cytokines and chemokines by interacting with the pattern recognition receptors (PRR) on the surface of epithelial cells to activate subsequent intracellular signaling pathways [3,4]. Due to the difficulties in isolating and cultivating the primary type I and II alveolar epithelial cells, almost all the functions of lung epithelial cells in innate immunity response against infection have been observed in lung carcinoma A549 cell line, which is type II-like lung epithelial cells [5]. In our previous studies, we reported that the C-type lectin receptor dectin-1 was expressed in A549 cells and mediated the activation of phospholipase D (PLD) and *A. fumigatus* internalization into lung epithelial cells. Nevertheless, *A. fumigatus* internalization was not fully blocked by anti-dectin-1 antibody [6], thus indicating that other cell membrane receptors might also be involved in regulating the internalization of *A. fumigatus* as well.

CR3, which is a member of the complement receptors family that belongs to the family of β2 integrins, comprises two subunits known as α_M_ (CD11b) and β_2_ (CD18). It is a versatile receptor present on many leukocyte subsets that mediate adhesion, chemotaxis, and phagocytosis by complement opsonization or complement-independent manner [7,8]. The CD11b subunit of CR3 has two distinct functional domains, I-domain and lectin-like domain [9]. Complement component C3 is the essential component for opsonization in serum. The iC3b, the degradation product of C3 can adhere to the surfaces of *A. fumigatus* conidia (opsonization) to promote phagocytosis of pathogens by binding to the I-domain of CR3 on host cells [10]. On the other hand, CR3 can also bind with β-glucans through its lectin-like domain to recognize un-opsonized yeast particles [11]. In most leukocytes, ligation of CR3 triggers the activation of Syk (spleen tyrosine kinase) inside the host cells through the ITAM (immunoreceptor tyrosine-based activation motif)-like motif, subsequently causing activation of downstream pathways which give rise to the phagocytic effect [12]. Another family of cytoplasmic nonreceptor protein tyrosine kinases, which is connected to CR3 signaling in many cell types, is the focal adhesion kinase (FAK) family, consisting of FAK and proline-rich kinase 2 (Pyk2) [13]. Following CR3 activation, FAK is autophosphorylated at Y397 permitting a low level of kinase activity and creating an SH2 binding site for proteins like the p85 regulatory subunit of PI3K and Src-family protein tyrosine kinases, mainly Src itself [14,15]. Thus far, it has not yet been clarified whether CR3 is expressed in lung epithelial cells and whether it regulates the *A. fumigatus* internalization into lung epithelial cells. Previous studies have suggested that CR3 mediates gonococcus and HIV-1 invasion of in primary cervical epithelial cells and human intestinal epithelial cells, respectively [16,17].

As a precursor of a phospholipid, phosphatidic acid (PA) is an important second messenger in the cells that has a critical role in many physiological events, such as vesicle-mediated transport, cytoskeleton organization, growth and differentiation [18]. Different phospholipid-metabolizing pathways have been shown to contribute to PA production. An important phospholipase, phospholipase D (PLD) hydrolyses primarily phosphatidylcholine (PC) to produce PA and phospholipase C (PLC) hydrolyses phosphatidylinositol lipids (PPIs) producing diacylglycerol (DAG), which can be subsequently phosphorylated to PA by DAG kinase [19]. Conversely, PA can also form DAG by phosphatidic acid phosphohydrolase (PAP) enzymes [20]. Previous studies have showed that PLD activation is an early event in β2 integrin-mediated phagocytosis in neutrophils [21] and both subtypes of PLD, PLD1 and PLD2 coordinately regulate macrophage phagocytosis [22]; however, the signaling pathway from CR3 to PLD activation is still poorly understood. In addition, the addition of PA partially restored fMLP-induced and CR3-mediated transepithelial migration, while PLD-derived PA was involved in changing the affinity of CR3 for its ligands in human eosinophils [23,24].

In the present study, we investigated the potential role of CR3 on *A. fumigatus* internalization into type II alveolar epithelial cells and its effect on host intracellular PA content induced by *A. fumigatus*.

## Results

### Expression of CR3 (CD11b/CD18) in airway epithelial cells

**Figure 1.**
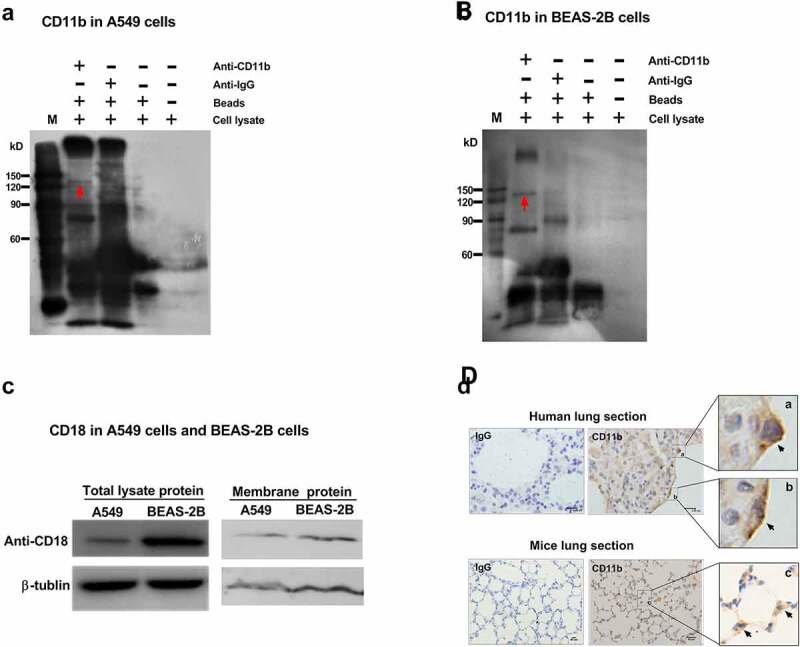
Expression of CR3 (CD11b/CD18) in airway epithelial cells. Total cell lysates of either A549 cells (a) or BEAS-2B cells (b) were incubated with protein A/G agarose beads and CD11b expression were analyzed by immunoprecipitation with anti-CD11b mAb (ab52478). The red arrows in A and B indicated the band of CD11b protein. (c) The total lysate protein and membrane protein of A549 cells and BEAS-2B cells were also analyzed for CD18 expression by immunoblotting using an anti-CD18 antibody (ab52920). β-tublin was used as a loading control. (d) Representative paraffin sections of human lung tissues from surgical specimens from seven lung cancer patients and mice lung tissues from normal C57BL/6 wild-type mice were stained for CD11b using anti-CD11b mAb (ab52478) and photographed at an optical magnification of 100. The arrows (black) indicated the staining of CD11b on lung epithelial cells. In human lung section, scale bar = 9.95 μm (IgG and CD11b). In mice lung section, scale bar = 50 μm (IgG and CD11b). a, b and c were the amplification of the specified area respectively. Images of immunohistochemical staining and immunoblots shown here are characteristic of 3 independent experiments

It is well known that CR3 is predominately expressed in phagocytes, such as neutrophils, macrophages, and eosinophils. To identify whether CR3 is also expressed in airway epithelial cells, the cell lysate of type II lung carcinoma epithelial cells A549 and immortalized bronchial epithelial cells BEAS-2B were enriched by immunoprecipitation with protein A/G agarose and analyzed by immunoblotting. As illustrated in (Figure 1(a)), the CD11b bands could be detected at the band of 130kDa by anti-CD11b antibody in A549 cells, as well as in BEAS-2B cells (Figure 1(b)). Meanwhile, CD18 could be directly detected in the total lysate proteins and membrane proteins of A549 and BEAS-2B cells (Figure 1(c)). Further, seven samples of human lung tissue from different patients and mice were collected, and CD11b was detected by immunohistochemical staining in lung epithelium (Figure 1(d)). The results showed that all of the samples were CD11b-positive (black arrow indicated). These data demonstrated that CR3 might be expressed in airway epithelial cells.

### C3 opsonization improves the internalization of conidia into alveolar epithelial cells

**Figure 2.**
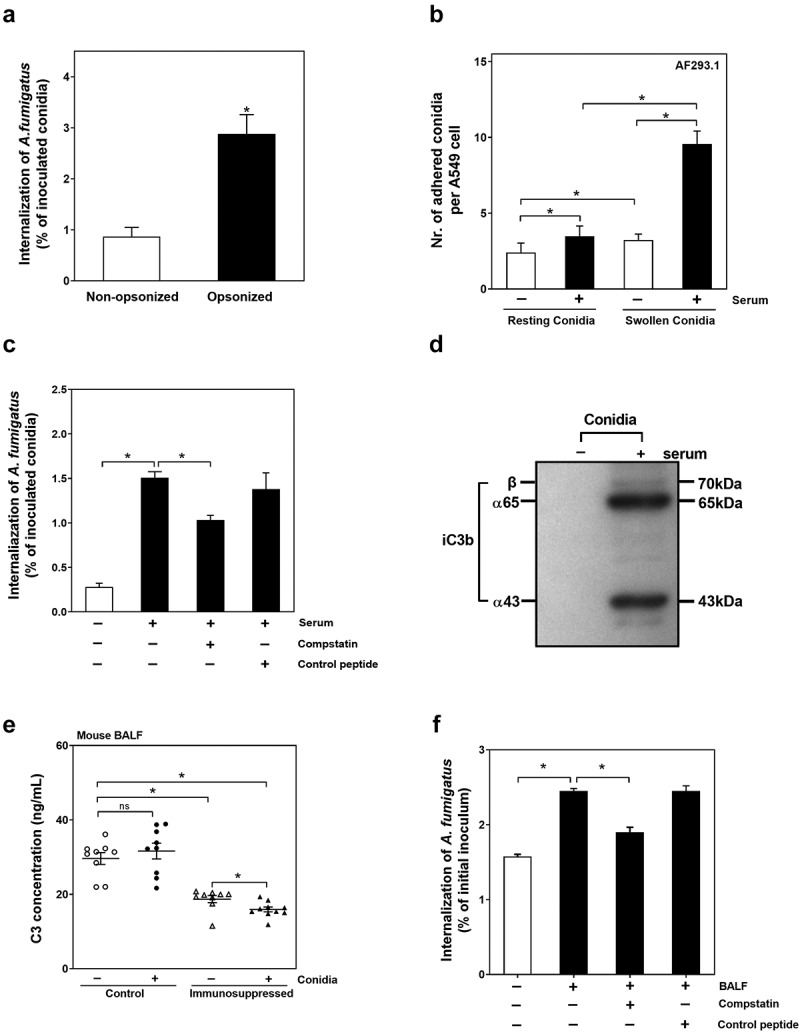
C3 opsonization improves the internalization of conidia into lung epithelial cells. (a) A549 cells were infected with the resting conidia of *A. fumigatus* ATCC13073 opsonized with or without human serum at an MOI of 10 for 6 h, and the nystatin protection assay was used to analysis ability of *A. fumigatus* internalization. (b) A549 cells were infected with the resting conidia and swollen conidia of *A. fumigatus* AF293.1 opsonized with or without human serum for 6 h, respectively. MOI = 10. Then the conida were stained by Calcufluor white. To analyze the adhesion of conidia to cells, 10 fields per coverslip were captured, and the number of adhered conidia per cell were determined. (c) The resting conidia of *A. fumigatus* ATCC13073 were pre-treated with DMEM (white column), human serum (the first black column), human serum plus C3 specific inhibitor compstatin (100 μM)(the second black column) or control peptide (100 μM)(the last black column), then added into A549 cells at an MOI of 10 for 6 h. The nystatin protection assay was used to analyze the ability of *A. fumigatus* internalization. (d) Resting conidia of *A. fumigatus* ATCC13073 were incubated with or without 10% human serum for 30 min at 37°C, and iC3b binding were detected by immunoblotting. The 65 kDa band is iC3b α65 chain, the 43 kDa band is iC3b α43 chain. Data are representative of three independent experiments. (e) The complement C3 present in mouse BALF samples were tested by complement C3 Mouse ELISA kit (Abcam). Control means the BALF samples from normal C57 mice infected or not infected by *A. fumigatus* ATCC13073 conidia. Immunosuppressed means the BALF samples from mice with suppressed immune function by hydrocortisone before infected or not infected by *A. fumigatus* ATCC13073 conidia. (f) The resting conidia of *A. fumigatus* ATCC13073 were pre-treated with DMEM (white column), mouse BALF (the first black column), mouse BALF plus C3 specific inhibitor compstatin (100 μM)(the second black column) or control peptide (100 μM)(the last black column), then added into mouse primary lung epithelial cells at an MOI of 10 for 6 h. The nystatin protection assay was used to analyze the ability of *A. fumigatus* internalization. Experiments are performed in 3 independent experiments. The multiple t-test was performed in the above results. ns (not significant), *p* > 0.05; *p < 0.05. Data is shown as means ± s.e.m

Since the conidia internalization into epithelial cells is a critical process for establishing of *Aspergillus* infection [25,26], we first checked the effect of serum opsonization on conidia internalization of *A. fumigatus* into lung epithelial cells. As shown in (Figure 2(a)), after pretreatment with 10% human serum these opsonized *A. fumigatus* conidia had significantly higher internalization rate compared to untreated conidia. Similar effect was also found when they were opsonized with 10% fetal calf serum (Figure S1(a)). Further, a uridine dystrophic strain of AF293, AF293.1, which is known to keep the dormant state in the absence of uracil, was used to study the effect of serum opsonization on conidia adhesion to A549 cells. As shown in (Figure 2(b)) and (Figure S1(b)), the number of adhered conidia on A549 cells significantly increased when the conidia were opsonized by serum regardless of they were resting or swollen. The swollen conidia opsonized with serum had the highest value of adhesion to A549 cells. Similar increasing trend was also observed regarding to the percentage of A549 cells adhering the AF293.1 conidia (Figure S1(c)). Taken together, opsonization of conidia by serum might improve conidia adhesion and internalization of *A. fumigatus* into A549 cells.

Given that the complement component C3 is a key component for opsonization in serum, the role of C3 on conidia internalization into A549 cells was further determined. Compstatin, a quencher of C3 was uesd to inactivate the C3. It was found that the conidia opsonized with compstatin-pretreated serum had a significantly lower level of internalization compared to the conidia opsonized with normal or control peptide-pretreated serum (Figure 2(c)). The deposition of iC3b on the surface of conidia was also verified (Figure 2(d)). The 65kDa and 43kDa bands were α65 chain and α43 chain of iC3b, respectively. The results indicated that iC3b is deposited on the surface of serum-opsonized conidia and participates in conidia internalization into A549 cells.

The complement C3 content in bronchoalveolar lavage fluid (BALF) of mice was also checked. As shown in (Figure 2(e)), the C3 content in BALF of immunosuppressed mice was much lower than that in immunocompetent mice. Interestingly, the infection by *A. fumigatus* conidia was significantly accompanied by with the decrease of C3 content in BALF of immunosuppressed mice but had no effect on C3 content in BALF of normal mice, which suggested that the small amount of C3 might be further consumed during infection process in the lung of immunosuppressed mice.

Further, the effect of C3 in BALF on conidia internalization was further checked. As shown in (Figure 2(f)), pretreatment of conidia with BALF of mice could significantly raise the conidia internalization into primary lung epithelial cells of mice; still, this increase in internalization was inhibited if the BALF of mice was preincubated with compstatin (100 μM) before conidia were added. In contrast, the preincubation of BALF with control peptide (100 μM) did not affect the promotion of BALF to the conidia internalization. These results demonstrated that C3 in serum and BALF is involved in conidia internalization of *A. fumigatus* into lung epithelial cells.

### Requirement of CR3 for efficient internalization of A. fumigatus conidia into alveolar epithelial cells

**Figure 3.**
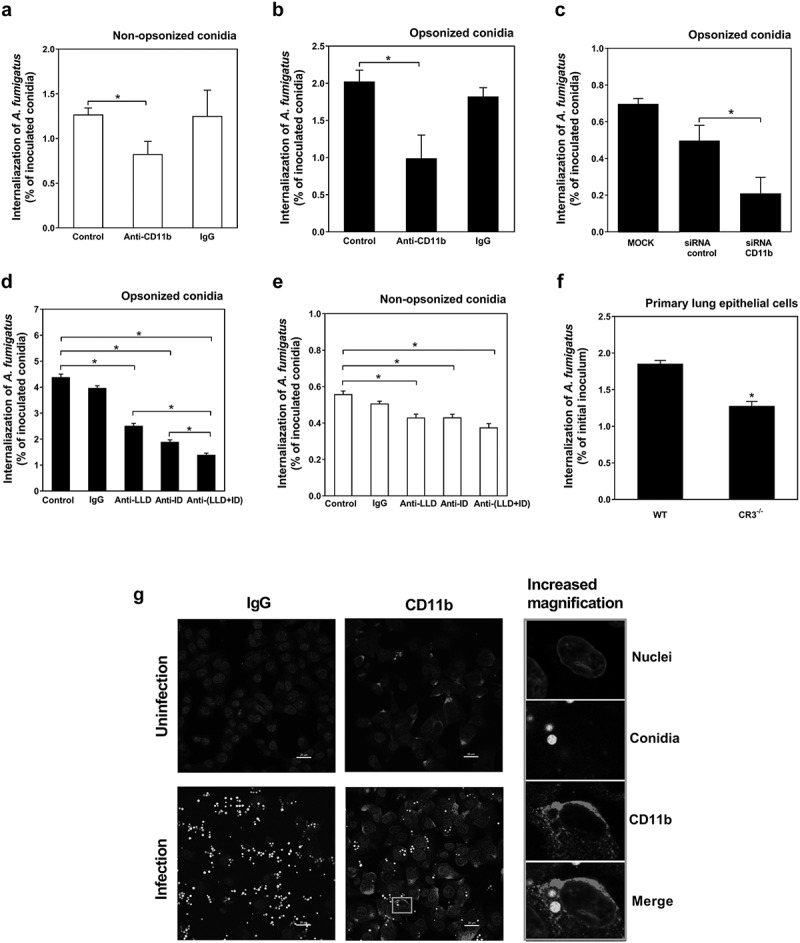
CD11b are involved in the internalization of *A. fumigatus* conidia into lung epithelial cells by opsonization. (a-f) The internalization of *A. fumigatus* ATCC13073 conidia into A549 cells was analyzed by nystatin protection assay, MOI = 10, incubation time = 6 h. A549 cells were pre-treated with or without anti-CD11b monoclonal antibody (NBP1-28,423, 10 μg/ml) and isotype control IgG for 30 min at 37°C, respectively, then infected with the resting conidia of *A. fumigatus* ATCC13073 which were opsonized without (a) or with (b) human serum. (c) A549 cells were transfected siRNA for negative control and CD11b by lipofectamin 2000 for 48 h, respectively, then infected with the resting conidia of *A. fumigatus* ATCC13073 which were opsonized with human serum. A549 cells were pre-treated with or without anti-CD11b either I-domain (anti-ID, 10 μg/ml) and/or lectin-like domain (anti-LLD, 10 μg/ml) monoclonal antibody and isotype control IgG for 30 min at 37°C, respectively, then infected with the resting conidia of *A. fumigatus* ATCC13073 which were opsonized without (d) or with (e) human serum. (f) Primary pulmonary epithelial cells from wild type C57 mice and CR3^−/-^ mice were stimulated with the resting conidia of *A. fumigatus* ATCC13073 for 6 h. Data were representative of 3–4 independent experiments or from means ± s.e.m of four independent experiment. The multiple t-test was performed, *p < 0.05. (g) The internalization of *A. fumigatus* into A549 cells and the expression of CD11b were monitored after staining by fluorescence microscopy using fluorescence microscope Olympus BX51 (green, *A. fumigatus* conidia; red, CD11b). A549 cells were infected with or without the resting conidia of *A. fumigatus* 13073 stably expressing green fluorescence protein (MOI = 10) for 6 h. Then the cells were stained with anti-CD11b primary antibody(ab75476) and Alexa Fluor 594 goat anti-rabbit IgG secondary antibody(red), DAPI (stain nuclei, blue). The images were processed with Image-Pro Express, Scale bar was shown on the images

CR3 has been demonstrated to mediate opsonic and non-opsonic phagocytosis of immunocytes regardless of binding with iC3b [27]. We further investigated whether CR3 was required for *A. fumigatus* conidia internalization into lung epithelial cells. The monoclonal antibody against CD11b (NBP1-28,423) was used to block the CR3 receptor. As shown in (Figure 3(a,b)), the blockade of CR3 of A549 cells by antibody resulted in a significant reduction of internalization into A549 cells by either non-opsonized or opsonized conidia. When the expression of CD11b was inhibited by siRNA for CD11b, the internalization of opsonized conidia was also clearly down-regulated compared to the groups transfected by either control siRNA (Figure 3(c)). Meanwhile, the function of CR3 on conidia adhesion to A549 cells was also detected by using the resting conidia AF293.1 cultured without uracil. It was shown that blockage of CR3 by anti-CD11b antibody did not affect the adhesion of non-opsonized resting conidia to A549 cells (Figure S2(a-c)) but inhibited that of serum-opsonized resting conidia (Figure S2(b-d)). These results indicated that CR3 might contribute to internalization of *A. fumigatus* conidia into lung epithelial cells via opsonization.

The two functionally different domains of CD11b, I-domain and lectin-like domain, can bind the complement component iC3b and β-(1,3) (1,6)-D-linked glucan [28,29], respectively, and mediate the phagocytosis of *A. fumigatus* conidia by phagocytes alone or both. For lung epithelial cells, blocking either I-domain or lectin-like domain could inhibit the internalization of non-opsonized or opsonized conidia. For the non-opsonized conidia, the blockage of both domains had no further inhibition on the internalization (Figure 3(d)), whereas for opsonized conidia inhibition of both domains, it had synergistic effect on conidial internalization (Figure 3(e)). These data showed that the two domains of CR3 might function differently in regulating *A. fumigatus* internalization by opsonization or non-opsonization mechanism.

To further characterize the role of CR3 during *A. fumigatus* conidia internalization, the primary pulmonary epithelial cells from wild-type mice and CR3^−/-^ mice were isolated and infected with the resting conidia of *A. fumigatus*. It was found that loss of CR3 significantly reduced the internalization of conidia into primary lung epithelial cells (Figure 3(f)). Moreover, CR3 deficient mice(CR3^−/-^) were more susceptible to *A. fumigatus* infection than wild-type mice(WT) (Figure S3). Furthermore, by incubating *A. fumigatus* resting conidia with A549 cells, it was observed under fluorescent microscopy that CD11b (red, immune-labeled) was enriched around the internalized conidia (GFP-tagged), thereby forming red-labeled rings (Figure 3(g)). Together, these results suggested that CR3 has an import role on the internalization of *A. fumigatus* conidia into lung epithelial cells.

### Regulation of swollen conidia-induced PA production in alveolar epithelial cells by CR3

**Figure 4.**
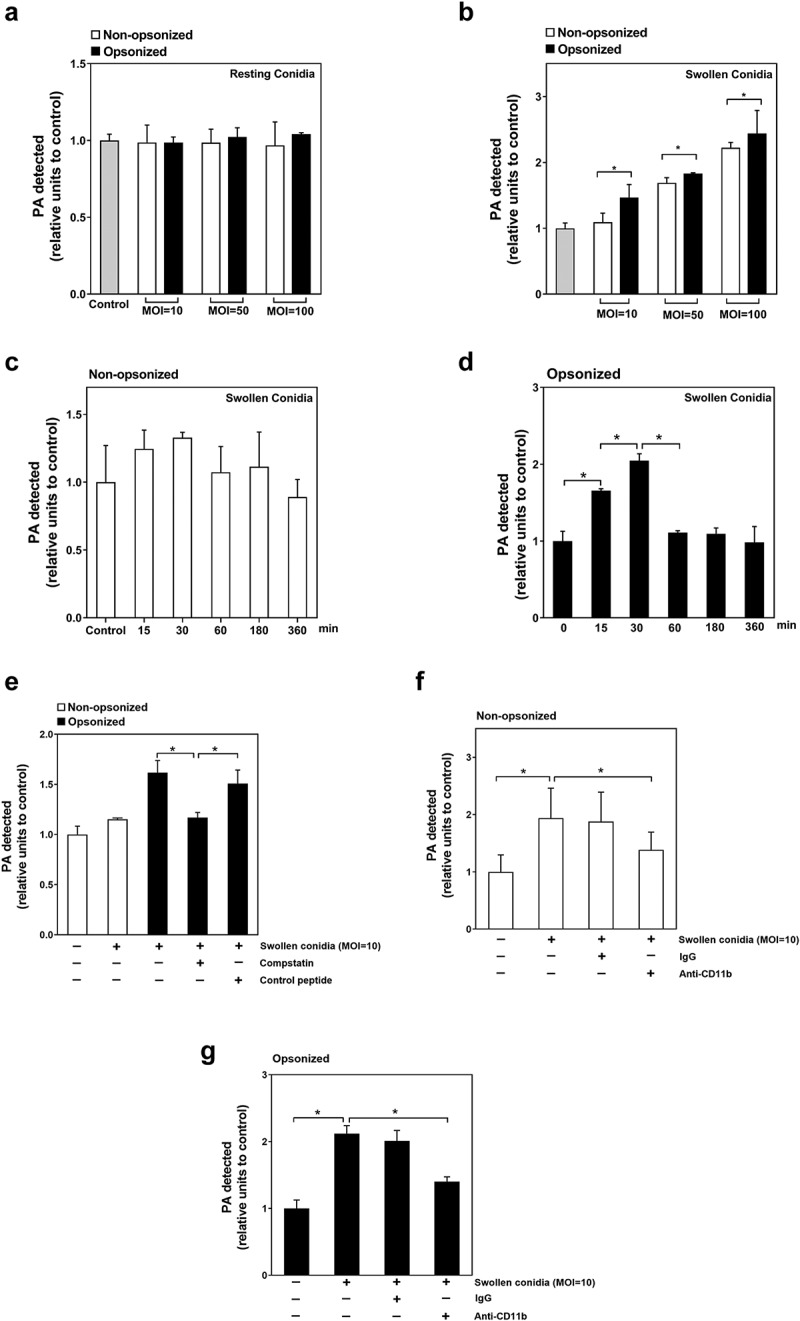
Regulation of swollen conidia-induced PA production in A549 cells by CR3. (a-g) the intercellular PA content was measured by an enzyme-coupled fluorometric assay. A549 cells were stimulated with *A. fumigatus* ATCC13073 resting (a) or swollen (b) conidia opsonized with or without 10% human serum at an indicated MOI for 1 h. (c, d) A549 cells were infected by *A. fumigatus* ATCC13073 swollen conidia (MOI = 10) opsonized without (c) or with (d) 10% human serum for different duration. (e) The swollen conidia of *A. fumigatus* ATCC13073 were opsonized with human serum, human serum plus C3 specific inhibitor compstatin (100 μM) or human serum plus control peptide (100 μM), then added into A549 cells at an MOI of 10 for 1 h. (f, g) A549 cells were infected with the swollen conidia of *A. fumigatus* ATCC13073 which were opsonized without (f) or with (g) 10% human serum in the presence or absence of anti-CD11b antibodies (NBP1-28,423, 10 μg/ml) for 30 min. Differences of the PA content in A549 cells which were stimulated with conidia in different experiment conditions were compared. Data are from mean ±s.e.m of three independent experiments. The multiple t-test was performed, *p < 0.05

As we previously demonstrated the significant PLD activation during *A. fumigatus* internalization into A549 cells [6], herein, we further investigated the potential regulation on cellular PA (product of PLD catalyzation) by CR3 during *A. fumigatus* internalization. First, the resting conidia could not induce the increase of cellular PA content in A549 cells even at MOI = 100 regardless of they were opsonized or not (Figure 4(a)). In contrast, swollen conidia induced significant increase of cellular PA content at MOI of 50 and 100 except 10 and the opsonization could obviously enhance this increase (Figure 4(b)). Moreover, at MOI of 10 the non-opsonized swollen conidia did not alter the intracellular PA contents of A549 cells at different time-points within 3 hours whereas the opsonized swollen conidia induced the increase of PA, which reached the highest level at 30 min post infection, then decreased and remained stable until 360 min (Figure 4(c,d)). These results hinted that opsonization significantly contributed to the cellular PA increase induced by swollen conidia, especially at lower MOI. It is more likely that internalization of conidia was correlated with an increase cellular PA rather than opsonization.

To demonstrate the involvement of complement C3 in cellular PA increase in A549 cells, C3-specific quencher, compstatin (100 μM) was used to pretreat the serum. It was found that swollen conidia opsonized by this pretreated serum could not improve the cellular PA level compared to that by normal serum or control-peptide pretreated serum (Figure 4(e)). Furthermore, blockade of CR3 by anti-CD11b antibody significantly reduced PA production in A549 cells induced by non-opsonized or opsonized swollen conidia (Figure 4(f,g)). These data indicated that C3 and its receptor CR3 might be involved in the cellular PA signal of lung epithelial cells induced by swollen conidia of *A. fumigatus*; however, involvement of some other element of conidia could not be excluded.

### *FAK is required for CR3-mediated* A. fumigatus *internalization and PA production*

Subsequently, we investigated the downstream signal molecule of CR3 during CR3-mediated *A. fumigatus* internalization and PA production in A549 cells. Since Syk has been reported as a critical downstream effector of CR3 receptor [12], the expression and function of Syk in A549 cells were first checked during conidia internalization. Surprisingly, the expression of Syk could not be detected in A549 cells in this study (Figure S4), which was in accordance with previous studies [30,31]. As CR3 is a member of integrin receptors, which mediates the phosphorylation of FAK kinase, another critical downstream molecule in the cells, the role of FAK in response to *A. fumigatus* infection was investigated. First, as illustrated in (Figure 5a), the resting conidia alone could not simulate the FAK phosphorylation in A549 cells but the opsonization by human serum could cause FAK phosphorylation. In contrast, swollen conidia alone could effectively induce FAK phosphorylation and their opsonization enhanced this phosphorylation. Further, the silence of expression of both CD11b and CD18 significantly inhibited the opsonized swollen conidia-induced FAK phosphorylation in A549 cells (Figure 5b).

**Figure 5a.**
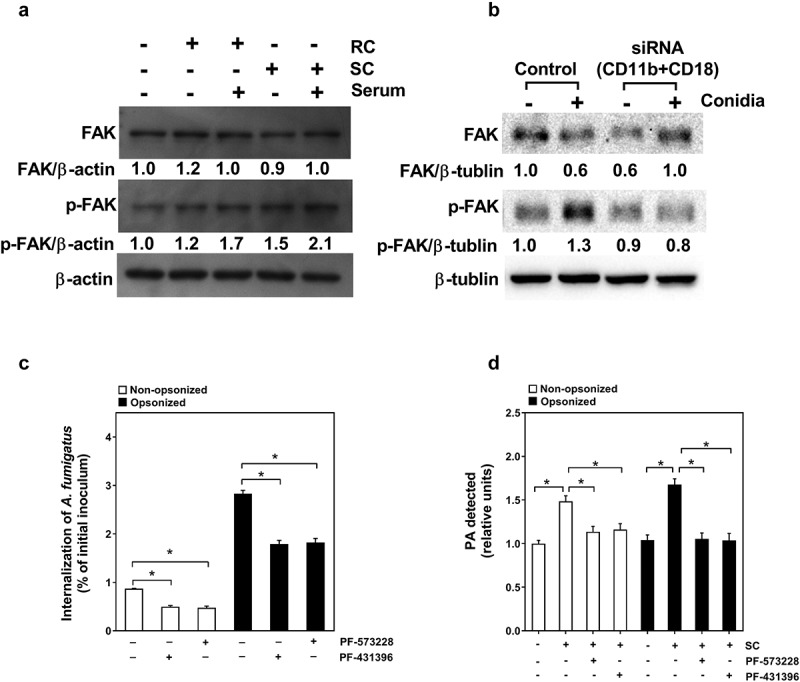
Regulation of swollen conidia-induced PA production in A549 cells by CR3. (a-g) the intercellular PA content was measured by an enzyme-coupled fluorometric assay. A549 cells were stimulated with *A. fumigatus* ATCC13073 resting (a) or swollen (b) conidia opsonized with or without 10% human serum at an indicated MOI for 1 h. (c, d) A549 cells were infected by *A. fumigatus* ATCC13073 swollen conidia (MOI = 10) opsonized without (c) or with (d) 10% human serum for different duration. (e) The swollen conidia of *A. fumigatus* 13073 were opsonized with human serum, human serum plus C3 specific inhibitor compstatin (100 μM) or human serum plus control peptide (100 μM), then added into A549 cells at an MOI of 10 for 1 h

**Figure 5b.**
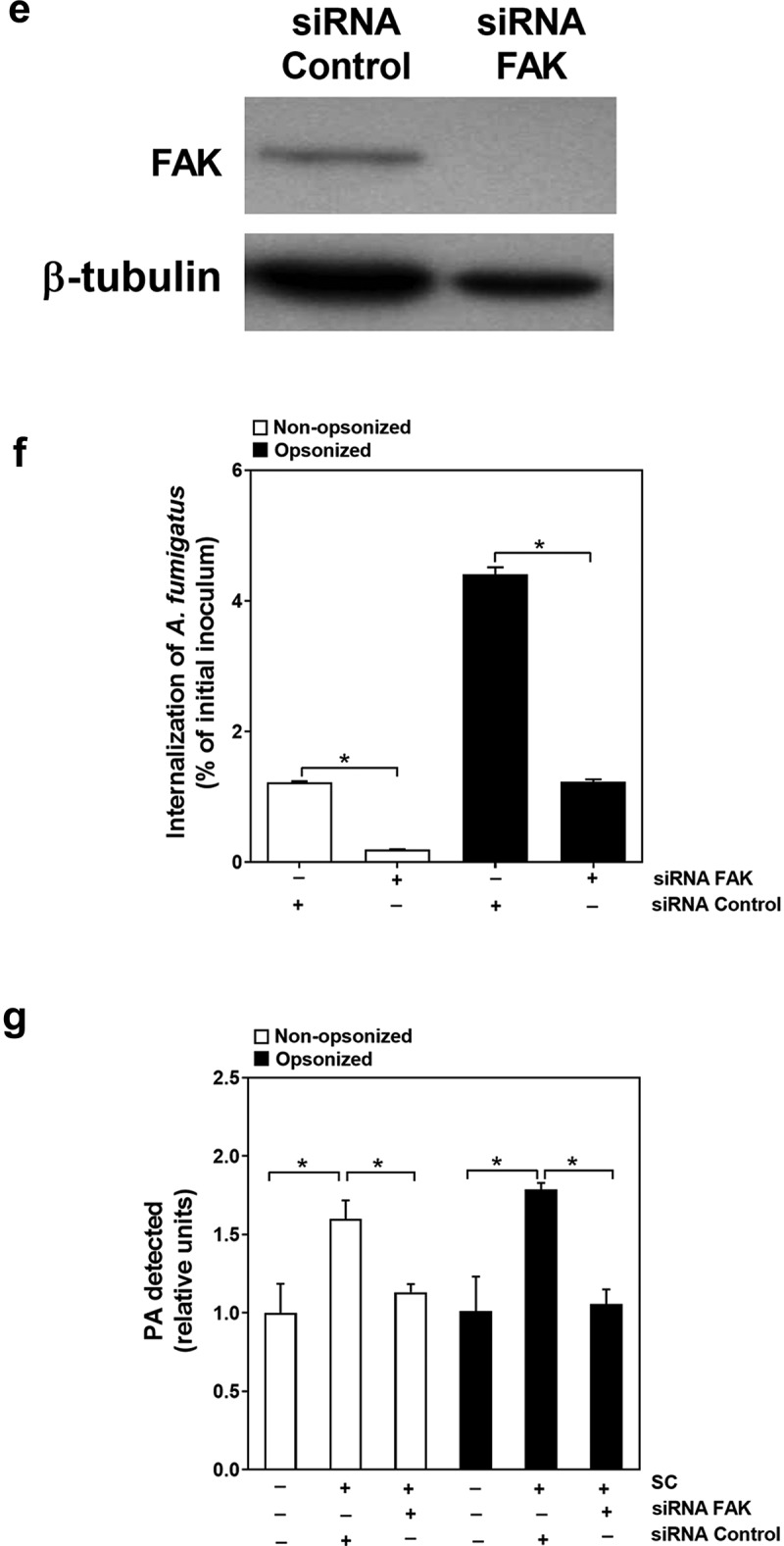
(f, g) A549 cells were infected with the swollen conidia of *A. fumigatus* ATCC13073 which were opsonized without (f) or with (g) 10% human serum in the presence or absence of anti-CD11b antibodies (NBP1-28,423, 10 μg/ml) for 30 min. Differences of the PA content in A549 cells which were stimulated with conidia in different experiment conditions were compared. Data are from mean ±s.e.m of three independent experiments. The multiple t-test was performed, *p < 0.05

We also checked the possible effect of FAK on conidial internalization and PA production in A549 cells. It was shown that pretreatment of A549 cells by specific inhibitors of FAK, PF-573,228 and PF-431,396, could efficiently reduce the internalization of the conidia and intercellular PA content of A549 cells (Figure 5a). Similarly, down-regulation of FAK expression by transfecting siRNA for FAK into A549 cells (Figure 5a) could simultaneously impress the internalization of the conidia and intercellular PA content of A549 cell (Figure 5b). Taken together, these data indicated that FAK might function as a downstream effector for CR3-mediated conidia internalization and PA production during *A. fumigatus* infection on alveolar epithelial cells.

## Discussion

Alveolar epithelial cells have a crucial role in both the maintenance of immune homeostasis and defense against pathogens in the lung [32]. To the best of our knowledge, this might be the first study reporting that CR3 was expressed in alveolar epithelial cells and that it regulated the internalization of *A. fumigatus* conidia into these cells.

Our data showed that *A. fumigatus* conidia internalized and adhered more efficiently into A549 cells, also known as type II alveolar epithelial cells, when they were opsonized with human serum. Given that this up-regulation of conidial internalization could be prohibited by compstatin, a quencher of C3 degradation product iC3b, it was speculated that CR3, the well-known host receptor recognizing iC3b, might participate in mediating the internalization. However, the expression and function of CR3 in lung epithelial cells were not well investigated and remained unclear. Previous studies demonstrated that CD11b was lacking in rat alveolar epithelial cells by immunoprecipitation and immunoblotting analysis of membrane proteins in rat primary alveolar epithelial cells [33]. Contrary, our data demonstrated that CD11b was evidently expressed in human A549 cells and airway epithelial cells BEAS-2B by immunoprecipitation and immunoblotting. It was also detected by immunohistochemical staining in human. And CD18 was evidently expressed in the membrane proteins of human alveolar epithelial cell line A549 and airway epithelial cell line BEAS-2B, though the membrane fractions include both plasma membranes and internal membranes of the cells. This discrepancy may be due to the different expression of CR3 between rat and human alveolar epithelial cells. In addition, it is known that human bronchoalveolar lavage fluid contains C3, thus pathogens inhaled into the airway of the host may be first opsonized by pathway activity that mediates the binding of C3 to the surface of pathogens, thereby activating the response of phagocytic cells [34]. Our data also showed that when *A. fumigatus* conidia was preincubated with mouse bronchoalveolar lavage fluid, the internalization of conidia into A549 cells significantly increased, and this increase was inhibited when mouse bronchoalveolar lavage fluid was pretreated by C3 quencher. These data may indicate that the inhaled *A. fumigatus* conidia could be opsonized by the C3 in bronchoalveolar lavage fluid, subsequently be swallowed by alveolar macrophages or internalized into the alveolar epithelial cells for further infection.

Interestingly, CR3 mediated *A. fumigatus* conidia internalization into alveolar epithelial cells in a complement-dependent or -independent manner. This was in line with the previous studies reporting that CR3 mediated phagocytosis under nonopsonic and opsonic conditions in nonphagocytic Chinese hamster ovary cells stably expressing the human CR3 [35,36]. It is well known that I-domain of CD11b, the α subunit of CR3, binds canonical ligands like extracellular matrix proteins, iC3b and intercellular adhesion molecule 1 (ICAM-1). On the other hand, the lectin-like domain of CD11b binds β-glucan, a (1,3) (1,6)-β-D-linked glucose polymer, that is a structural component of fungal cell wall and functions as a fungal pathogen-associated molecular pattern (PAMP). Previous studies have shown that leukocyte function is differentially regulated upon ligation of CR3 at the I-domain, the lectin-like domain, or both [9,37]. In our study, blocking I-domain or lectin-like domain significantly inhibited the internalization of non-opsonized or opsonized conidia. It seemed puzzling that blocking I-domain inhibited the internalization of non-opsonized conidia that should bind to lectin-like domain. There are two possible explanations for this result, one of which is that A549 cells can be induced to secrete inflammatory factors and complement components, such as C3, that may promote clearance of pathogenic microbes via A549 cells. The concentration of iC3b (degradation products of C3) in culture supernatant significantly increased after swollen conidia of *A. fumigatus* infection in the presence of human serum (Figure S5), and iC3b bound to conidia surface in the presence of human serum (Figure S6). The other explanation might be that the blockade of I-domain may hinder the binding of lectin-like domain with β-glucan, thus leading to insufficient priming of downstream signaling pathway for internalization. Furthermore, blocking both of the two domains had synergistic effect on inhibiting the internalization of opsonized conidia, but not non-opsonized conidia. These results indicated that co-occupancy of the I-domain and lectin-like domain by iC3b and polysaccharides in fungal cell walls, such as β-glucan, might induce a greater increase of internalization of opsonized conidia into A549 cells. However, a lectin-like domain may predominantly control the internalization of non-opsonized conidia into host cells, so that blocking another ligation domain might not further inhibit conidial internalization.In addition, CR3 deficient mice(CR3^-/-^) were more susceptible to *A. fumigatus* infection than wild type mice(WT) (Figure S3). However, this does not directly prove the roles of CR3 in the alveolus. These would need either conditional CR3 KO or BM chimera mice to conform the anti-*A.fumigatus* infection roles of CR3 in the alveolus.As a signal phospholipid, PA is a key factor in cellular membrane dynamic and related physiological events, like endocytosis, vesicle-mediated transport, cytoskeleton organization, growth, and differentiation [18]. It has been demonstrated that CR3-mediated phagocytosis is regulated by PLD, which hydrolyzes the most abundant membrane phospholipid phosphatidylcholine (PC) to produce PA [21]. The phagocytosis of iC3b-opsonized immune complexes by human neutrophils may be attributable to CR3-mediated PLD activation [38] and the translocation of CD11b onto the cell surface is also PLD-dependent [39]. PLD-derived PA was involved in changing the affinity of CR3 for its ligands in human eosinophils [23,24]. We previously demonstrated that *A. fumigatus* swollen conidia could stimulate PLD activity in A549 cells [6]; however, cellular PA level was not absolutely and positively correlated with PLD activity as PA can be produced by many other metabolic pathways [40]. In the present study, ligation of CR3 could significantly induce the PA production following *A. fumigatus* infection and opsonization by serum can further augment intracellular PA level stimulated by swollen conidia. In contrast, resting conidia were not able to induce intracellular PA production regardless if they were opsonized or when MOI rose to 1:100. These results indicated that an opsonization-independent way for PA regulation existed i.e., the exposed molecules on the surface of conidia during germination may stimulate increase in PA level, which predominantly occurs through CR3 in A549 cells during the internalization of *A. fumigatus*. We also checked the role of PLD in regulating PA level and found that inhibition of PLD could not decrease the PA level induced *A. fumigatus* internalization (data not shown), which suggested that PA synthesis might mainly be controlled by other productive pathways, such as diacylglycerol kinases (DGK) or phosphatidic acid phosphatase (PAP).

Another intriguing finding was that FAK, but not Syk, functioned as downstream effector of CR3 to mediate the conidial internalization and PA production in lung epithelial cells. Syk is a major cellular kinase for integrin “outside-in” signals that can modulate the activity of FAK kinase [41,42]. However, it was rather surprising that little or no expression of Syk in either A549 cells or BEAS-2B cells was detected in present study (Figure S4). This result was not in line with some earlier reports [43] but it supported the findings that very little Syk existed in primary alveolar type II epithelial cells with FACS isolation, RT-PCR and western blot confirmation [44]. With reference to FAK kinases, thus far, two major FAK family kinases, FAK and Pyk2, were extensively studied in cell adhesion signaling downstream of integrin activation [45]. Pyk2 is mainly expressed in the nervous system and T-cells whereas FAK is widely distributed in most cells of the body [46]. In the present study, we provided evidence that FAK might response to CR3 signal to regulate conidial internalization and cellular PA production in alveolar epithelial cells. Anyway, the involvement of other tyrosine kinases in Src-family kinases, such as Hck, Fgr, Lyn, and Src, could not be excluded and further investigation is necessary.

Moreover, the integrin α5β1 on epithelial cells might mediate the invasion of *A. fumigatus* by interacting with a thaumatin-like protein CalA on the surface of *A. fumigatus* cell wall [47]. Recently, it was reported that another β-glucan-recognizing receptor, EphA2, is also involved in the internalization of *A. fumigatus* conidia during infection and might be modulated by DHN-melanin. However, dual blockade of EphA2 and Dectin1 did not completely inhibit the internalization of conidia, which hinted at a possible involvement of other receptors during internalization [48]. All these findings, including CR3 in this study, suggested a complex interaction during *A. fumigatus* internalization into epithelial cells. In this process, structure and components of *A. fumigatus* conidia seem to dynamically change during swelling and germination, which might also induce a different receptor usage for adherence and internalization at the lung cell surface under physiological circumstance. Certainly, the detailed regulation patterns need to be further elucidated.

In summary, our results demonstrated that CR3 is definitely expressed in alveolar epithelial cells and has an important role on *A. fumigatus* invasion in alveolar epithelial cells through FAK activation. Our findings shed new light on the innate immune response of epithelial cells to fungal infection in the lung, while further regulation mechanisms in alveolar epithelial cells against *A. fumigatus* infection remains to be defined.

## Methods and materials

### A. fumigatus *strains, cell line*

*A. fumigatus* ATCC13073 constitutively expressing green fluorescent protein was generously provided by Dr. Margo Moore (Simon Fraser University, Burnaby, BC, Canada). *A. fumigatus* AF293.1(pyrG-) was a gift from Dr. Wei Liu (Peking University First Hospital, Beijing, China). *A. fumigatus* CEA17 Δ*ku80* was a gift from Prof. Gustavo H. Goldman (Hans-Knoell-Institut, Jena, Germany). *A. fumigatus* Δ*pksP* (Recipient strain was CEA17 Δ*ku80*) was constructed at our laboratory. All *A. fumigatus* strains were propagated on Sabouraud dextrose agar (10 g/l peptone, 40 g/l glucose, and 15 g/l agar) for 5 ~ 8 d at 37°C. Except where indicated, *A. fumigatus* ATCC13073 was used for all experiments. The type II human alveolar epithelial cell line A549 and human airway epithelial cell line BEAS-2B were obtained from ATCC and cultured in DMEM and RPMI-1640(GIBCO, Germany), respectively, supplemented with 10% fetal calf serum (GIBCO, Germany), 100 U/ml streptomycin, and 100 U/ml penicillin at 37°C in a humidified 5% CO_2_ incubator.

### Preparation of conidia

*A. fumigatus* conidia were harvested and prepared as described in a previous study. Briefly, after 5 ~ 8 d of culture, *A. fumigatus* conidia were dislodged from agar plates by gentle washing and resuspended in sterile phosphate-buffered saline supplemented with 0.1% Tween 20 (PBST). The conidia were then passed through 8 layers of sterile gauze to remove hyphal fragments and enumerated on a hemacytometer. Swollen conidia with early germling with <5 μm hyphal extensions were prepared by incubating resting conidia in liquid sabouraud dextrose agar for 4–6 h at 37°C 120rpm. Fungal cells were washed twice and stored at 4°C for use.

### Reagents, siRNAs and antibodies

Human serum was obtained from healthy adult volunteers, divided into aliquots, and stored at **−8**0°C until being used. Calcofluor White Stain(18,909) and Phorbol-12-myristate-13-acetate (PMA) (p1585) were purchased from Sigma-Aldrich. C3-specific inhibitor compstatin (ICVVQDWGHHRCT) and control peptide (IAVVQDWGHHRAT) were from Tocris Bioscience (Bristol, UK). siRNAs for negative control (SR30004), CD11b (SR302467), and FAK (SR303877) were purchaesd from Origene. FAK inhibitors PF-573,228 (869,288–64-2, USA) and PF-431,396 (HY-10,460, USA) were purchased from MedChemExpress. The list of antibodies used in the study as well as their specificities, applications, and source, are shown in the (Table 1).

**Table 1.** Discription of antibodies used in the study

Destination	Cat. No.	Discription	Specificity	Applications	Source
Anti-CD11b	NBP1-28,423	Mouse monoclonal [M1/70]	Human CD11b	Blocking	Novus
LM 2/1	BMS104	Mouse Monoclonal Antibody (LM2/1)	Human CD11b (I-domain)	Blocking	eBioscience
VIM 12	CD11B00	Mouse Monoclonal Antibody (VIM12)	Human CD11b (Lectin-like domain)	Blocking	Invitrogen
Anti-CD11b	ab64347	Rat monoclonal [M1/70] to CD11b	Human CD11b (I-domain)	Blocking	Abcam
IgG2	14–4724-82	Mouse IgG2a kappa Isotype Control	Isotype control	Blocking	eBioscience
IgG1	SA1-12,182	Mouse IgG1 Isotype Control	Isotype control	Blocking	Thermo Scientific Pierce
Anti-CD11b	ab52478	Rabbit monoclonal	Human CD11b (I-domain)	IHC-P, IP, WB	Abcam
Anti-CD18	ab52920	Rabbit monoclonal	CD18 phosphorylated on Serine 745	WB	Abcam
Anti-iC3b	A209	Mouse monoclonal	A murine monoclonal antibody to a neo-epitope expressed on iC3b	WB	Quidel
Anti-β-actin	sc-47,778	Mouse monoclonal	Total levels of β-actin protein	WB	Santa Cruz
IgG	Zdr-5307	HRP-conjugated goat anti-mouse	The secondary antibody	WB	ZSGB-BIO
IgG	Zdr-5306	HRP-conjugated goat anti-rabbit	The secondary antibody	WB	ZSGB-BIO
Anti-CD11b	ab75476	Rabbit monoclonal	Mouse CD11b aa 250–350 (internal sequence)	IF	Abcam
IgG	1,400,412	Alexa Fluor 594 goat anti-rabbit	The secondary antibody	IF	Life technologies
Anti-FAK	3285	Rabbit polyclonal	Endogenous levels of FAK protein	WB	CST
Anti-p-FAK	3281	Rabbit polyclonal	Endogenous levels of FAK only when phosphorylated at tyrosine 576/577	WB	CST
Anti-Syk	22,206-1-AP	Mouse polyclonal	Endogenous levels of Syk protein	WB	Proteintech

### Serum opsonization and inhibition experiments

Conidia were opsonized by 10% fetal calf serum or 10% human serum for 30 min at 37°C. For blocking the CR3 receptor, A549 cells were incubated in DMEM containing anti-CD11b antibodies or isotype control antibodies for the indicated dose for 30 min. To inhibit C3 activities, compstatin (100 μM) or control peptide (100 μM) was added to serum and cellular culture supernatant. Next, the preparations were used for the internalization, adhesion or the PA assays.

### *Analysis of* A. fumigatus *internalization*

The nystatin protection to determine the *A. fumigatus* internalization into A549 cells was performed as previously described. Briefly, A549 cells were seeded at 2 × 10^4^ cells/well in 96-well plates (Corning) and grown for 16 h, after which they were pretreated with nonspecific PLD inhibitors, antibodies, or transfected by different plasmids. The cells were incubated with the live resting conidia of *A. fumigatus* at an MOI of 10 at 37°C for 6 h in 5% CO_2_. Subsequently, the wells were washed three times with PBST and incubated with nystatin (20 μg/ml) in DMEM for 3 h at 37°C. The monolayers were washed twice with PBST and lysed with 0.25% Triton X-100 for 15 min. The released conidia were diluted and plated onto SDA agar (3 replicate plates/well) and incubated at 37°C for 24 h. The colonies were counted to determine the total intracellular conidia. The internalization capacity was expressed as a percentage of the initial inoculum.

### Analysis of conidial adhesion to cells

Adhesion studies were performed as previously described. Briefly, A549 cells were incubated with *A. fumigatus* conidia for 6 h washed three times with PBS, fixed in 4% paraformaldehyde, and then incubated 5 min with Calcufluor white at a 1:2 ratio with 10% KOH. After washing twice with PBS, cells were examined by differential interference contrast (DIC) and fluorescence microscopy. To analyze the adhesion of conidia to cells, 10 fields per coverslip were captured, and the adhered conidia were determined.

### Determination of iC3b deposition on the conidial surface

The iC3b deposition on the conidial surface was determined by the following assay [49]. Briefly, a total of 1 × 10^8^ conidia per 100 μl were incubated with DMEM, A549 culture supernatant or 10% human serum for 30 min at 37°C. The conidia were washed three times with 0.01% SDS-50 mM Tris (pH 7.1)-10 mM EDTA before SDS extraction. Conidia surface proteins were extracted by boiling for 5 min in 2× sample buffer (10% SDS, 0.25 M Tris [pH 6.8], 0.5 M DTT, 0.5% bromphenol blue, 50% glycerol). The conidial extracts were submitted to SDS-PAGE and transferred to a PVDF membrane, probed with mouse anti-iC3b (1:2000), and then visualized with HRP-conjugated anti-mouse secondary antibody (1:5000).

### Enzyme immunoassay for the quantitation of iC3b

The concentrations of iC3b in different reaction buffers were measured by with an enzyme immunoassay kit (A009, Quidel) according to the manipulation instruction. In brief, 100 μl of each diluted specimen was added to its assigned microassay well and incubated at room temperature for 30 min. After being washed, the microassay wells were added with 50 μl iC3b conjugate and incubated at room temperature for 30 min. Then, each well was added with 100 μl freshly prepared Substrate Solution and incubated at room temperature for 30 min. Finally, 50 μl Stop Solution was added to each well to stop the enzymatic reaction. The absorbance reading was determined at 405 nm in a plate reader (Spetra Max M5, Molecular Device, USA) and iC3b concentrations were analyzed based on the standard curve.

### PA determination

The levels of cellular PA were measured using an enzyme-coupled fluorometric assay, as previously described. Briefly, A549 cells (1 × 10^6^ cells/dish) were cultured in 35 mm cell culture dishes (Corning) at 37°C overnight in DMEM without fetal calf serum. Then, the cells (alone or pretreated with antibodies) were incubated with or without *A. fumigatus* conidia (MOI = 10) for 30 min at 37°C. Incubations were terminated by removing the reaction liquids and adding 500 ul ice-cold methanol to each dish. Cells were scraped and added with 250 μl 1 M NaCl and 500 μl ice-cold chloroform [50]. The mixture was then thoroughly mixed and centrifugated at 3,400 rpm for 10 min at 4°C. The lower lipid phase was collected and dried by Termovap Sample Concentrator (MD200, sample concentrator). The samples were dissolved in 1% Triton X-100 aqueous solution and added with 80 µl of Reagent 1 containing 10,000 units/ml lipase (Sigma), 50 mM NaCl, 50 mM Tris-HCl (pH7.4), and then were incubated at 37°C for 1 h. After the incubation, the lipase was heat-inactivated at 96°C for 3 min, and the denatured enzyme was precipitated by centrifugation (7, 200 g, 5 min). The supernatant (50 μl/well) was added to 96-well black plates (Corning), and incubated with 50 μl of Reagent 2 (5 units/ml GPO (Sigma), 5 units/ml peroxidase (Sigma), 300 μM Amplex Red (Invitrogen), 0.2% Triton X-100, 40 mM NaCl, and 40 mM Tris-HCl (pH7.4)) at room temperature for 30 min, and was then incubated with 20 μl Amplex Red Stop Reagent (Invitrogen) for 30 min. This resulted in the production of the fluorescent reporter resorufin in a PA-concentration-dependent manner, as measured by fluorescence emission at 590 nm after excitation at 544 nm in a plate reader (Spetra Max M5, Molecular Device, USA).

### Immunofluorescence analysis and immunoblotting

A549 cells (5 × 10^4^ cells/ml) were seeded onto coverslips in 24-well plates and grown for 24 h. The ATCC13073 resting conidia opsonized with 10% human serum for 30 min were added to the wells to start the invasion for 6 h. To stop internalization, the cells were washed three times with PBS and fixed in ice-cold 70% methanol in PBS buffer for 15 min at 4°C. The cells were then permeabilized for 5 min with 0.1% Triton X-100 in PBS buffer and were incubated with 5% goat serum for 30 min at room temperature, after which they were stained with the indicated antibodies. The primary antibody used was anti-CD11b (ab75476), and the secondary antibody was Alexa Fluor 594-conjugated goat anti-rabbit IgGs. The preparations were observed with a fluorescence microscope Olympus BX51. Green fluorescence was captured with a 515- to 540-nm band pass filter, and red fluorescence was captured with a 590- to 610-nm band pass filter. The images were processed with Image-Pro Express.

For immunoblotting, equal amounts of protein from cell lysates were separated by SDS-PAGE and transferred to a PVDF membrane. Then, the membrane was incubated at room temperature for 3 h or at 4°C for overnight with the different primary antibody according to experimental needs and was subsequently incubated at room temperature for 1–2 h with the HRP-conjugated secondary antibody. The proteins were visualized by enhanced chemiluminescence (Santa Cruz Biotechnology Inc.). A densitometric analysis of the immunoblots was performed with Image J2x (Wayne Rasband, National Institutes of Health, USA).

### Immunohistochemical staining

Seven samples of human lung tissue were collected from different patients and fixed in 10% (vol/vol) formaldehyde. The lung tissues were embedded in paraffin and sectioned. After a series of treatment, tissue sections were treated with 5% BSA, incubated for 3 h with anti-CD11b primary antibody (ab52478) and subsequently incubated for 3 h with the HRP-conjugated secondary antibody. Images were taken with a BX51 microscope (Olympus), Olympus DP71 camera using brightfield illumination at 400× magnification. These images were later processed using Image Pro Express 6.0 (Media Cybernetics Inc., MA, USA).

### In vivo *virulence assay*

This study was carried out in accordance with the recommendations of Management of Laboratory Animal, Laboratory Animal Welfare and Ethics Committee, Academy of Military Medical Sciences, China. The protocol was approved by the Laboratory Animal Welfare and Ethics Committee (IACUC-13-2016-002), Academy Military Medical Sciences, China. C57BL/6 mice were obtained from the Animal Center of Academy of Military Medical Sciences. CR3^−/-^ mice were purchased from the Nanjing Biomedical Research Institute of Nanjing University. The mouse model of invasive pulmonary aspergillosis was established using a protocol described by Sugui et al. [51], which involves immunosuppression with steroids, with modification. Ten- to twelve-week-old male C57BL/6 mice were treated with hydrocortisone acetate (5 mg/mouse, administered subcutaneously) every other day, beginning on day – 4 relative to infection and ending on day +4, for a total of 5 doses. For inoculation, the mice were anesthetized with isoflurane, after which 5 × 10^6^ conidia in 25 μl of PBS containing 0.1% Tween-20 was injected intranasally. For a collection of bronchoalveolar lavage fluid (BALF) from mice, after 4 d of infection, the mice were anesthetized with 25% ethyl carbamate and the skin and muscles of the neck were cut off, exposing the trachea, after which the bronchoalveolar lavage fluid was extracted. Then, 1 ml of sterile PBS was slowly injected into the trachea by syringe and pumping back liquid. This was repeated three times, putting the extracted liquid into a 1.5 ml sterile EP tube, performing centrifugation for 10 min at 2500rpm, and collecting the supernatant.

For survival analysis, the mice were observed every day after infection for 1 month. For histopathological examination, the lung tissue sections obtained from mice from each group were dissected, fixed in 10% (vol/vol) formaldehyde, and stained with hematoxylin and eosin (H&E) and periodic acid-Schiff (PAS). Images were taken using a BX51 microscope (Olympus) with an Olympus DP71 camera using brightfield illumination at 200× magnification. These images were later processed using Image Pro Express 6.0 (Media Cybernetics Inc., MA, USA).

### Statistical analysis

The data shown in the figures are from a representative experiment or from a mean ± standard error of the mean (s.e.m) of 3–4 independent experiments which were performed in triplicate. Except where otherwise indicated, a student’s paired t-test was used to compare the differences between the groups, using GraphPad Prism8.0.2 software. Values of *p* < 0.05 were considered to be statistically significant.

## Supplementary Material

Supplemental Material

## Abbreviations

CR3 Complement receptor 3

BALF Bronchoalveolar lavage fluid

PA Phosphatidic acid

FAK Focal adhesion kinase

Syk Spleen tyrosine kinase

PPR Pattern recognition receptors

PLD Phospholipase D

ITAM Immunoreceptor tyrosine-based activation motif

Pyk2 Proline-rich kinase 2

PLC Phospholipase C

PPIs Phosphatidylinositol lipids

DAG Diacylglycerol

PAP Phosphatidic acid phosphohydrolase

ICAM-1 Intercellular adhesion molecule 1

MOI Multiplicity of infection

PBST Phosphate-buffered saline supplemented with 0.1% Tween 20

PMA Phorbol-12-myristate-13-acetate
